# Investigating STLV-1 infection in African green monkeys: a model for understanding HTLV-1 pathogenesis

**DOI:** 10.3389/fmed.2025.1616406

**Published:** 2025-06-30

**Authors:** Víctor Â. Folgosi, Liliane A. Carneiro, Sabri S. Sanabani, Felipe B. Freitas, Mayara N. S. da Silva, Amanda L. Silva, Gerlane N. Noronha, Ariela S. Farias, Hebert F. Culler, Carlos F. Apoliano, Luciano Lopes, Juliana Pereira, Luís Alberto P. C. Lage, Igor B. Costa, Camila M. Romano, Shirley V. Komninakis, Jorge Casseb

**Affiliations:** ^1^School of Medicine, University of São Paulo, São Paulo, Brazil; ^2^National Center for Primate (CENP), Belém, Brazil; ^3^Evandro Chagas Institute, Ananindeua, Brazil; ^4^Division of Bioinformatics and Biomedical Data Science, Federal University of São Paulo, São Paulo, Brazil

**Keywords:** HTLV-1, STLV-1, ATLL, adult T-cell leukemia/lymphoma, *Chlorocebus aethiops*

## Abstract

**Introduction:**

Simian T-cell leukemia virus type 1 (STLV-1) and human T-lymphotropic virus type 1 (HTLV-1) are homologous viruses with high genetic identity. STLV-1 infections in non-human primates serve as valuable models to study HTLV-1 pathogenesis.

**Methods:**

This study investigated STLV-1 infection in captive green monkeys (*Chlorocebus aethiops*) in Brazil. Blood samples from 52 animals were collected and analyzed for viral presence, genetic characterization, and pathological manifestations.

**Results:**

STLV-1 infection was detected in seven animals, corresponding to a seroprevalence of 13.4%. Phylogenetic analysis showed that the STLV-1 strains identified are more closely related to baboon STLV-1 strains and human African HTLV-1 isolates than to other STLV-1 variants, suggesting a shared evolutionary history and possible cross-species transmission. Clinically and hematologically, STLV-1 infection in C. aethiops presented parallels to HTLV-1 infection in humans, including the presence of characteristic “flower cells” and similar lymphoproliferative disorders.

**Discussion:**

These findings reinforce the relevance of *C. aethiops* as a natural model for studying HTLV-1 infection and pathogenesis. The genetic and clinical similarities indicate potential mechanisms of viral evolution and transmission, providing insights that may aid in understanding HTLV-1-associated diseases in humans.

## Introduction

Human T-lymphotropic virus type 1 (HTLV-1) infection is a major global health problem, affecting an estimated 10–20 million people worldwide, with a particularly high prevalence in certain geographic regions such as southwestern Japan, the Caribbean, parts of South America, sub-Saharan Africa and the Middle East (1). As the first human retrovirus discovery in 1980, HTLV-1 has been extensively studied since them, revealing its etiologic role in adult T-cell leukemia/lymphoma (ATL) and HTLV-1-associated myelopathy (HAM), as well as various inflammatory diseases (2–4). Despite decades of research, there remain critical gaps in our understanding of HTLV-1 pathogenesis, transmission dynamics and effective therapeutic approaches, largely due to the limitations of existing experimental models.

The search for appropriate animal models to study HTLV-1 infection has led researchers to investigate its simian counterpart, Simian T-cell leukemia virus type 1 (STLV-1). STLV-1 shares remarkable genetic identity with HTLV-1, with nucleotide sequence identity ranging from 90% to 95% depending on the viral region examined (5). This close genetic relationship has led to the hypothesis that HTLV-1 originated from multiple cross-species transmission events of STLV-1 from non-human primates to humans, most likely through hunting activities and direct contact with infected animals, followed by the subsequent dissemination of the virus, primarily facilitated by the transatlantic slave trade to Latin America (6–8). The evolutionary relationship between these viruses provides a unique opportunity to study retroviral zoonotic transmission patterns and the adaptations required for successful cross-species infection.

Simian T-cell leukemia virus type 1 has been detected in over 30 non-human primate species across Africa and Asia, with infection rates varying significantly between species and geographical locations (9, 10). Among Old World monkeys, the genera *Cercocebus*, *Cercopithecus*, and *Lophocebus albigena* stand out. Despite the potential of the genus *Chlorocebus aethiops*, especially *C. aethiops*, as a model for retroviral infections due to its susceptibility and ease of handling, there remains a significant gap in the study of STLV-1 infection in these species (11–13). Due to the fact that the alternative species to the use of Rhesus monkeys, being of management relatively easy and high prolificacy in captivity (14, 15). These monkeys are widely distributed throughout sub-Saharan Africa and have been introduced to various locations worldwide, including the Caribbean islands and parts of South America, through historical human activities (16, 17).

Brazil offers a unique setting for investigating STLV-1 and HTLV-1 interactions due to several converging factors. The country has one of the largest populations of HTLV-1-infected individuals worldwide—estimated at 800,000 to 2.5 million (18)—and hosts multiple primate facilities with captive *C. aethiops* and other non-human primate species. Brazil is also among the few countries to maintain research colonies of *C. aethiops*, enabling controlled studies of STLV-1 in Old World monkeys within the same geographic region as endemic HTLV-1 infections. This configuration supports comparative research on transmission, pathogenesis, and viral evolution that is not feasible elsewhere.

The pathogenesis of HTLV-1 in humans is marked by complex virus-host interactions that can lead to a wide range of clinical outcomes. Although most infected individuals remain asymptomatic, approximately 2%–5% progress to adult T-cell leukemia/lymphoma (ATL), and 0.25%–3.8% develop HTLV-1-associated myelopathy/tropic (HAM) (19, 20). The mechanisms underlying disease progression are not fully understood but are believed to involve a combination of viral factors, host genetic background, and environmental influences (21). Similarly, studies in non-human primates (NHPs) naturally infected with STLV-1 have revealed the development of lymphoproliferative diseases that closely resemble ATL in humans.

For instance, d’Offay et al. (22) reported STLV-1-associated T-cell lymphomas in baboons, characterized by clonal expansion of infected T cells and immune activation during tumor development. Likewise, Miura et al. (23) demonstrated that Japanese macaques naturally infected with STLV-1 exhibit clinical and molecular features analogous to HTLV-1 infection in humans, supporting their use as a relevant model for studying viral pathogenesis and host responses.

A hallmark feature HTLV-1 and STLV-1 infection is the presence morphological changes in infected lymphocytes, named “flower cells” – atypical lymphocytes with lobulated nuclei resembling flower petals (24). These cells serve as a diagnostic marker for HTLV-1 infection in humans and have been observed in STLV-1-infected non-human primates, suggesting shared pathogenic features (23). Both viruses primarily target CD4+ T lymphocytes and persist through mitotic spread (proliferation of infected cells) and *de novo* infection via cell-to-cell contact (25).

Phylogenetic analyses of HTLV-1 and STLV-1 strains have identified distinct molecular subtypes (genotypes) with specific geographical distributions. HTLV-1 is classified into seven major subtypes (a–g), with the Cosmopolitan subtype (HTLV-1a) being the most widespread globally (1). Similarly, STLV-1 strains exhibit genetic diversity corresponding to their host species and geographical origins. The phylogenetic relationships between these viruses provide valuable insights into their evolutionary history and the patterns of cross-species transmission events that have shaped their current distribution (9, 26, 27).

Understanding HTLV-1 pathogenesis is critical for developing effective preventive and therapeutic strategies, but progress has been limited by the lack of animal models that fully reflect the human disease. Although models such as transgenic and humanized mice have been used, they fall short in replicating the complex virus-host dynamics of natural infection (28, 29). In contrast, STLV-1-infected non-human primates offer greater physiological relevance but require thorough characterization to confirm their utility.

In this context, we investigated STLV-1 infection in a captive *C. aethiops* population in Brazil, where HTLV-1 is endemic. Using phylogenetic, clinical, and hematological analyses, we aimed to clarify the evolutionary relationship of these STLV-1 strains with known HTLV-1/STLV-1 isolates and assess the pathological features of infection.

## Materials and methods

This is a cross-sectional study conducted at the Laboratory of Medical Investigation 56 (LIM/56), Faculty of Medicine, University of São Paulo. The study included *C. aethiops* specimens that have been maintained in captivity for over three generations at the National Primate Center (CENP) in Belém, Pará, where they remain under standardized conditions, including regular veterinary care, environmental enrichment, and controlled diet and housing.

### Sample collection and study population criteria

Peripheral blood samples were collected from each of the 52 animals via femoral vein puncture. Species identification was performed on site by visual inspection, following the criteria established in the Kingdon Field Guide to African Mammals (30). Blood samples were centrifuged at 2,500 rpm for 15 min to separate the plasma and leukocyte layer (buffy coat). Both plasma and buffy coat fractions were then stored at −20°C for subsequent analyses.

Inclusion criteria: Only non-human primates of the genus *Chlorocebus* were included in this study.

Exclusion criteria: Only animals older than 2 years were included to avoid potential interference from maternal antibodies.

### Clinical and hematologic profiling

Each *C. aethiops* specimen underwent a standardized clinical evaluation, including physical examination and hematological analysis. The physical examination included measurement of body weight, inspection of the ocular and oral mucosa, assessment of skin turgor for hydration status, palpation of lymph nodes and auscultation of the lungs. In addition, a complete blood count was performed to determine the hematologic parameters. Peripheral blood smears were taken and examined microscopically to detect atypical and multilobulated lymphocytes characteristic of HTLV-1-associated T-cell leukemia, such as “flower cells.”

### Serological test

To quantify the titers of cross-reactive antibodies against human T-cell lymphotropic virus type 1 (HTLV-1) in all plasma samples, serological testing was performed using the Western blot method (HTLV-Blot 2.4, MP Biomedicals). Briefly, plasma samples were diluted 1:100 in the provided sample buffer and incubated on nitrocellulose strips pre-coated with HTLV-1 viral antigens, including disrupted virions, the recombinant gp21 (GD21) protein, and specific peptides such as MTA-1 (corresponding to residues 162-209 of the gp46 protein). Strips were incubated for 1 h at room temperature with gentle agitation. After washing to remove unbound antibodies, strips were incubated with horseradish peroxidase-conjugated anti-primate IgG secondary antibody for 30 min. Following further washes, antigen-antibody complexes were visualized using a chromogenic substrate according to the kit protocol.

Samples were classified as seropositive if they exhibited antibody reactivity against the p19 and p24 core antigens as well as envelope glycoproteins GD21 and MTA-1. Samples showing antibodies against GD21 with or without p19 but lacking p24 reactivity were considered inconclusive. Samples without any specific bands, with only non-specific bands, or exhibiting antibodies against structural proteins without the presence of GD21 or MTA-1 were classified as seronegative.

### Molecular studies

For the extraction of genomic DNA from buffy coat cells, the PureLink™ Genomic DNA Mini Kit (Invitrogen, Massachusetts, United States) was used, strictly following the manufacturer’s protocol. The quality and concentration of the extracted DNA were assessed using a Nanodrop spectrophotometer (Thermo Fisher Scientific, Massachusetts, United States).

For the detection of STLV-1 provirus, two pairs of specific primers amplifying regions of the LTR gene were used in a nested PCR approach. In the first amplification cycle, the forward (5′-TGACAATGACCATGAGCCCCAA-3′) and reverse (5′-CGCGGAATAGGGCTAGCGCT-3′) primers were employed. For the second cycle, a second pair of primers internal to the initial reaction was used: forward (5′-GGCTTAGAGCCTCCCAGTGA-3′) and reverse (5′-GCCTAGGGAATAAAGGGGCG-3′), generating a final amplicon of 645 base pairs. The nested-PCR reactions were prepared in a final volume of 26 μl, containing 11.45 μl of ultrapure H_2_O, 1.25 μl of 10 × reaction buffer, 3.0 μl of MgCl_2_ (50 mM), 6.0 μl of dNTPs (10 mM), 0.5 μl of each primer (forward and reverse, 20 pmol), 0.3 μl of Taq DNA polymerase, and 3.0 μl of genomic DNA. The thermocycling conditions included an initial denaturation at 94°C for 5 min, followed by 35 cycles of denaturation at 94°C for 30 s, annealing at 52°C for 30 s, and extension at 72°C for 40 s, with a final extension at 72°C for 10 min. The second amplification cycle used identical conditions with the internal primer pair.

### Sequencing

The identity and integrity of the amplified LTR gene fragments were confirmed by Sanger sequencing. Following nested PCR amplification, the resulting amplicons (645 base pairs) were purified using the PureLink™ PCR Purification Kit (Thermo Fisher Scientific), to remove excess primers, nucleotides, and enzymes. Sequencing reactions were carried out using the BigDye™ Terminator v3.1 Cycle Sequencing Kit (Applied Biosystems). Both forward and reverse primers from the nested PCR were used in independent reactions to ensure bidirectional coverage and increase accuracy.

Post-reaction cleanup of sequencing products was performed using the BigDye™ XTerminator™ Purification Kit to remove unincorporated dyes and salts. The purified reactions were then loaded onto an ABI 3130xl Genetic Analyzer (Applied Biosystems), and electropherograms were analyzed using standard capillary electrophoresis conditions. Sequencing quality was evaluated using Sequence Analysis Software, and only high-quality reads were considered for further alignment and phylogenetic analysis.

### Phylogenetic analyses

Phylogenetic analysis was carried out using the Maximum Likelihood (ML) method implemented in IQ-TREE (31). The most appropriate nucleotide substitution model, TIM2 + F + G4, was selected using the ModelFinder tool, which evaluates models based on the Akaike Information Criterion (AIC) and the Bayesian Information Criterion (BIC). This step ensured that the model best fit the evolutionary characteristics of the dataset. To evaluate the robustness of the inferred phylogenetic tree, a bootstrap analysis with 1,000 replicates was performed. Branches with bootstrap support values greater than 80% were considered statistically significant, providing confidence in the topology of the tree.

## Results

### Serological and molecular detection

**FIGURE 1:**
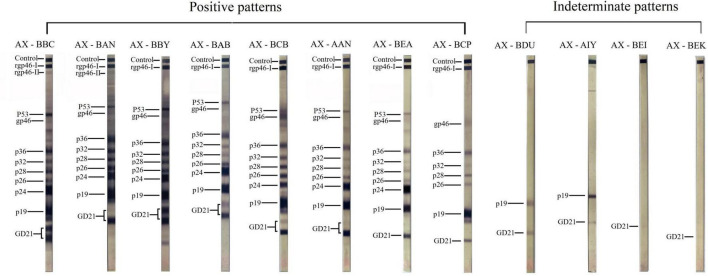
Western blot analysis of simian T-cell leukemia virus type 1 (STLV-1) serological profiles in non-human primates. Western blot results showing positive patterns (left) and indeterminate patterns (right) for STLV-1 seroreactivity in non-human primates. Specimens classified as positive exhibited strong reactivity to key STLV-1 proteins, including gp46, p19, and p24. Indeterminate samples displayed atypical profiles, with reactivity limited to GD21 and/or p19.

Among 52 sera specimens tested, 8 (15.4%) demonstrated robust serological responses against critical HTLV-1 proteins (gp46, p19, and p24), confirming STLV-1 infection. PCR amplification successfully detected proviral DNA in 7 (13.4%) of these seropositive specimens, while one case was confirmed exclusively through Western blot assay. Four additional specimens exhibited atypical immune profiles - two reacting solely to p19 and GD21, and two responding only to GD21 (Figure 1). These specimens, lacking proviral DNA detection and displaying non-standard serological patterns, were classified as indeterminate and excluded from subsequent analyses.

### Clinical characteristics

All seven PCR-confirmed STLV-1-positive animals were adult females with an average body weight of 3.5 ± 0.56 kg. Clinical assessment revealed three animals with dermal dehydration and two with lymphadenopathy, while none displayed respiratory or mucosal abnormalities.

### Hematological findings

**FIGURE 2:**
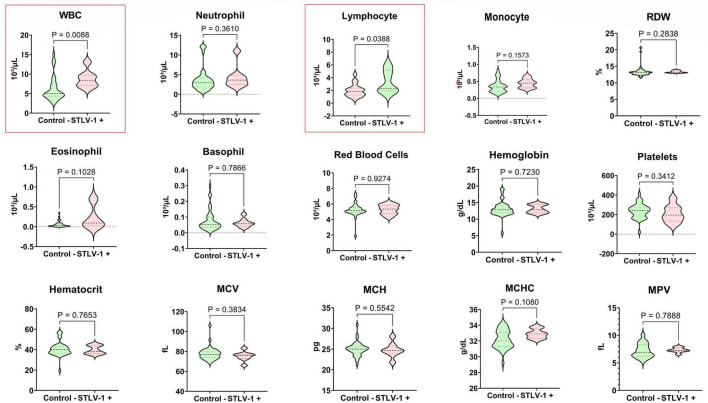
Comparative analysis of hematological parameters between *C. aethiops* STLV-1+ and healthy specimens. The comparison between non-parametric continuous variables was performed using the Mann-Whitney test.

**FIGURE 3:**
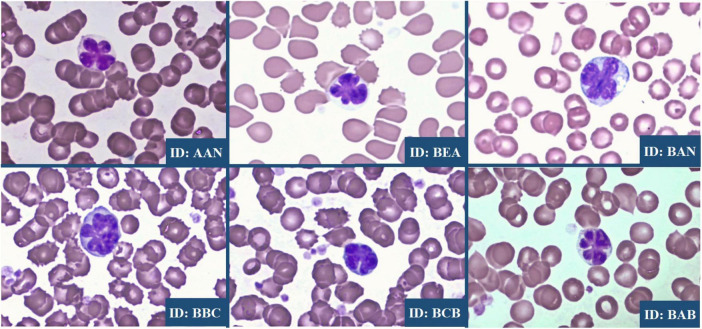
Flower cells observed in *C. aethiops* STLV-1+ specimens. The image shows a multilobed atypical cell with a characteristic “flower-like” appearance, as highlighted in the stained smear preparation.

Simian T-cell leukemia virus type 1-positive primates exhibited significant alterations in immune parameters, particularly elevated white blood cell counts with pronounced lymphocytosis (Figure 2). This pattern reflects the clonal expansion of infected T-cells typically observed in retroviral infections and mirrors findings in human HTLV-1 cases. Microscopic examination of blood smears from all positive specimens revealed the presence of distinctive atypical lymphocytes with multilobulated nuclei, known as “flower cells,” which are pathognomonic of HTLV-1-associated leukemia in humans (Figure 3). No other significant abnormalities in red blood cell morphology or platelet counts were detected in STLV-1-positive animals.

### Evolutionary relationships of STLV-1 isolates

**FIGURE 4:**
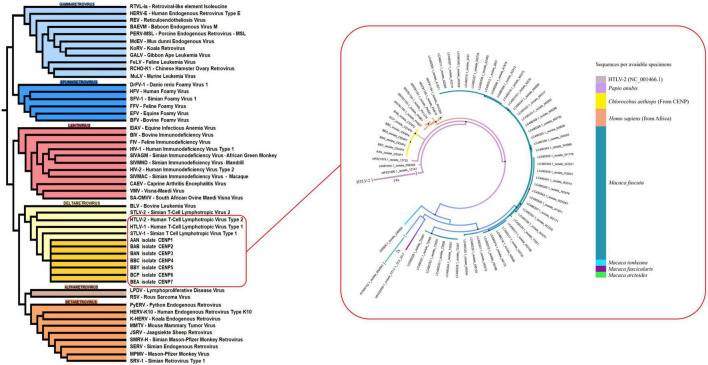
Phylogenetic analysis of simian T-cell leukemia virus type 1 (STLV-1) isolates highlighting the evolutionary relationship between *C. aethiops* and other primate hosts. The phylogenetic tree illustrates the evolutionary relationships among STLV-1 isolates, with *C. aethiops* represented by yellow branches. The analysis was performed using the 5′ 88–3′ 734 regions (645 bp, REF: MF621980.1) of the isolates. Black dots indicate branches with bootstrap support > 80. Phylogeny was inferred using Maximum Likelihood in IQ-TREE, with the TIM2 + F + G4 model selected by ModelFinder according to AIC/BIC, and branch support assessed by bootstrap. The distinct clustering of *C. aethiops* isolates (yellow) between baboon (purple) (*Papio anubis*) and human isolates (orange) (MN781150.1-MN781155.10) suggests a potential interface in cross-species transmission or a shared evolutionary history among these primate hosts.

Phylogenetic analysis revealed that STLV-1 isolates from *C. aethiops* form a distinct monophyletic cluster, clearly differentiated from previously described lineages (Figure 4). This cluster positions strategically among predominant viral lineages from African and Japanese origins.

Of note, these *C. aethiops* STLV-1 isolates demonstrate remarkable genetic proximity to STLV-1 strains from baboons (*Papio anubis*) (MF621980.1, MF621979.1, JX987040.1). More intriguingly, they occupy an intermediate evolutionary position between baboon isolates and HTLV-1 strains isolated from humans of African descent (MN781150.1 to MN781155.1). This human comparison group includes viral samples from West and North African populations as well as individuals from French Guiana with African ancestry.

The strategic intermediate phylogenetic position of the *C. aethiops* STLV-1 isolates suggests they may represent an ancestral form to contemporary human HTLV-1 strains, providing crucial evidence for understanding the evolutionary history and cross-species transmission dynamics of these retroviruses.

## Discussion

In our study, STLV-1 infection in captive *C. aethiops* in Brazil was investigated by serological, molecular, clinical and hematological analyzes to characterize the evolutionary relationships between the viruses and pathological manifestations in a region where HTLV-1 is endemic in humans. Our serologic and molecular results are consistent with established HTLV-1/STLV-1 studies while providing new insights into retroviral infection patterns through the identification of characteristic “flower cells” in all STLV-1-positive samples from this particular *C. aethiops* population in Brazil. The seroprevalence rate of 13.4% observed in our study is consistent with previously reported STLV-1 infection rates in various Old World primates, particularly the range of 11%–25% documented by Ishikawa et al. (32) in Indonesian macaques and the seroprevalence of approximately 25% in Japanese monkeys (33).

The significant serologic responses against critical HTLV-1 proteins (gp46, p19, and p24) in our positive samples reflect the antigenic similarity between STLV-1 and HTLV-1 and confirm the proven close evolutionary relationship between these retroviruses. Previous studies (34–36) on the timing of cross-species transmission provide a link to our results by providing an evolutionary context for the antigenic similarities we observed. The demonstration of strong cross-reactivity between STLV-1-infected monkey samples and HTLV-1 test antigens confirms molecular conservation between these viruses despite 27 300 years of separate evolution (37). This conservation explains why our diagnostic tests (EIA and WB) developed for HTLV-1 were successful in detecting infection in monkeys, validating our methodological approach.

Our observation that all PCR-positive STLV-1 animals were adult females is consistent with the epidemiologic patterns reported by Hayami et al. (33), who found that STLV-1 incidence in Japanese monkeys increases with age and is higher in females than in males. This pattern has been confirmed in several species, as infection status in Japan is positively correlated with age and the incidence of the disease is higher in females than in males. This sex-specific disparity is consistent with the epidemiology of HTLV-1 and suggests similar transmission dynamics in non-human primates. The positive correlation between STLV-1 infection status and age likely reflects cumulative exposure risk over time, as older animals have more opportunities for virus transmission through social interactions and grooming behaviors.

The higher incidence of disease in females compared to males can be attributed to several factors, including the more frequent grooming behavior of females, sex-specific immune responses that influence susceptibility to infection (38), maternal transmission routes, and social structures in which females remain in natal groups while males disperse. This pattern mirrors HTLV-1 epidemiology in humans, where seroprevalence generally increases with age and adult females have higher rates than males, particularly in endemic regions where maternal transmission is important (39, 40).

While most STLV-1-positive subjects in our study appeared clinically healthy on examination, a subgroup exhibited subtle clinical manifestations, especially skin desiccation and lymphadenopathy. These mild symptoms are in contrast to the more severe HTLV-1-associated diseases observed in humans, such as ATL and HAM. The high proportion of asymptomatic STLV-1 carriers in our population reflects the well-established pattern of HTLV-1 infection in humans, where approximately 90%–95% of infected individuals remain clinically silent throughout their lives. The absence of overt disease in most seropositive animals reflects the characteristically long latency period of deltaretrovirus infections, during which viral persistence can last for years or decades before pathologic consequences, if any, occur. These results suggest that our captive *C. aethiops* population provides a valuable model for studying the natural history of HTLV-1 infection, particularly the asymptomatic carrier state, which is the most common clinical outcome in humans.

Our hematologic findings showed an increased white blood cell count with lymphocytosis in STLV-1-positive animals, which is consistent with the immune response to viral infection (41). However, it would be premature to call this a clonal expansion in the absence of molecular evidence of T-cell clonality. Although HTLV-1 infection in humans can lead to clonal expansion of infected T cells, particularly during progression to ATL, this process usually occurs over decades and is not necessarily present in asymptomatic carriers (42, 43). The observed lymphocytosis probably represents a polyclonal immune response to viral antigens rather than a true clonal expansion.

In particular, the detection of “flower cells” in all positive samples is a critical finding, as these cells are pathognomonic for HTLV-1-associated leukemia in humans. This observation is consistent with previously published reports showing that STLV-1 induces ATL in naturally infected NHPs (44, 45), despite some differences in auxiliary proteins (7). The presence of these characteristic hematologic markers in our *C. aethiops* population argues that STLV-1-infected African green monkeys are a valuable model for HTLV-1 research. This is particularly important as African green monkeys have been identified as natural carriers of STLV-1 (37). The detection of flower cells in our samples represents a direct cellular manifestation that occurs in both monkey and human infections, emphasizing the translational value of this model.

Phylogenetic analysis reveals a clear relationship between STLV-1 isolates of *C. aethiops* and other primate hosts. The distinct monophyletic cluster formed by STLV-1 isolates occupies an intermediate position between baboon isolates (*Papio anubis*) and HTLV-1 strains from humans of African descent. This intermediate position provides convincing evidence for the evolutionary history of these retroviruses. The close genetic proximity between STLV-1 from *C. aethiops* and STLV-1 strains from baboons (MF621980.1, MF621979.1, JX987040.1) is consistent with previous findings by Meertens et al. (46), who identified STLV-1 in *C. aethiops* that was identical to an STLV-1 strain from *Papio anubis* (PAN 503) from the same geographic region in Cameroon. This suggests possible cross-transmission between different primate species living in the same environment.

The phylogenetic positioning of *C. aethiops* STLV-1 between baboon isolates and African-origin human HTLV-1 strains (MN781150.1 to MN781155.1) supports the hypothesis that these viruses may represent an ancestral lineage to modern HTLV-1 strains in humans. This finding is consistent with the hypothesis of multiple cross-species transmission from non-human primates to humans. As highlighted in the review by Jégado et al. (37), Yamashita et al. (47), the diversity of STLV-1 across various non-human primate species and its correspondence to specific HTLV-1 subtypes from the same geographic regions strongly suggest repeated zoonotic transmission events. This is particularly relevant considering that HTLV-1 subtype B is closely related to STLV-1 strains infecting various African primates, including chimpanzees and gorillas from central African regions, with sequence identity reaching 98% (48).

The transmission dynamics between STLV-1 and HTLV-1 are particularly noteworthy. In Japanese macaque troops infected with STLV-1, extremely high genomic identity (> 99%) has been observed (49), in contrast to the greater genetic diversity seen among African non-human primates and humans. his disparity suggests the influence of distinct evolutionary pressures or transmission patterns across geographic regions. While HTLV-1 is primarily transmitted among humans via sexual contact, mother-to-child transmission, or exposure to infected blood, STLV-1 transmission in non-human primates occurs mainly through aggressive interactions. However, sexual transmission may play a more prominent role in certain species, such as vervet monkeys (50, 51).

The molecular clock analysis performed by Dooren et al. (52) suggests that STLV-1 was introduced to the Asian continent approximately 156,000–269,000 years ago, while the origin of HTLV-1 subtypes A, B, D and E dates back to approximately 27,300 ± 8,200 years ago. These time frames in conjunction with the phylogenetic positioning of the STLV-1 isolates of *C. aethiops* contribute to our understanding of the evolutionary history of these retroviruses and their cross-species transmission patterns.

Despite these results, this study has several limitations. The small sample size of captive *C. aethiops* in a non-native environment may not fully represent wild populations, which could affect the generalizability of our results. The cross-sectional design prevents assessment of disease progression over time, limiting our understanding of the natural history of STLV-1 infection. In addition, we relied on diagnostic tests optimized for the detection of HTLV-1 in humans rather than STLV-1-specific tests, and molecular characterization was limited to partial viral sequences rather than complete genomes. The lack of immunophenotyping and T-cell receptor gene rearrangement studies prevents definitive confirmation of clonality in the observed lymphocytosis.

In conclusion, our results demonstrate that STLV-1 infection in captive *C. aethiops* produces serologic, molecular, and cellular manifestations remarkably similar to those of HTLV-1 infection in humans, with the phylogenetic positioning of these isolates providing compelling evidence for their role in the evolutionary pathway between simian and human deltaretroviruses. The presence of characteristic “flower cells” in all positive samples despite asymptomatic clinical presentations suggests that these animals may serve as valuable models for studying the early stages and natural history of HTLV-1 infection, particularly the mechanisms underlying the transition from asymptomatic transmission to disease development. This study supports the evidence for multiple cross-species transmission events in the evolutionary history of these retroviruses and emphasizes the importance of continuous monitoring of STLV-1 in non-human primates as potential reservoirs for emerging human pathogens.

Despite these advances, several questions remain unanswered. First, the temporal dynamics of clinical progression in STLV-1–infected primates, including the possible evolution toward diseases similar to ATL or HAM, have yet to be established due to the cross-sectional design of the study. Additionally, the absence of comprehensive analyses of cellular clonality, such as immunophenotyping and T-cell receptor rearrangement studies, limits the understanding of the true nature of the observed lymphocytosis. Another relevant point is the lack of complete genomic characterization of the isolated viruses, which restricts detailed analysis of genetic variations that may influence pathogenesis and transmissibility.

## Data Availability

The datasets presented in this study can be found in online repositories. The names of the repository/repositories and accession number(s) can be found in the article/supplementary material.
